# Progressive Retinal Vascular and Neuronal Degeneration in BXD32 Mice: A Model for Age-Dependent Neurovascular Pathology

**DOI:** 10.3390/ijms26199289

**Published:** 2025-09-23

**Authors:** Fan Xia, Shuizhen Shi, Seth E. Buscho, Erick Palacios, Melinda McCarty, Monia Nazemi, Lu Lu, Wenbo Zhang, Hua Liu

**Affiliations:** 1 Department of Ophthalmology and Visual Sciences, University of Texas Medical Branch, 301 University Boulevard, Galveston, TX 77555, USA; faxia@utmb.edu (F.X.); shushi@utmb.edu (S.S.); seth.buscho@bswhealth.org (S.E.B.); palacioskcire@gmail.com (E.P.); mmnazemi@utmb.edu (M.N.); we2zhang@utmb.edu (W.Z.); 2 Department of Genetics, Genomics and Informatics, University of Tennessee Health Science Center, Memphis, TN 38163, USA; mmccarty@uthsc.edu (M.M.); llu@uthsc.edu (L.L.); 3 Department of Neurobiology, University of Texas Medical Branch, Galveston, TX 77555, USA

**Keywords:** retinal vasculature, BXD32, OCT, OCTA, dysfunction, degeneration

## Abstract

Retinal vasculature is essential for maintaining visual function by supporting metabolically active neurons. However, the retina lacks redundant blood supply, rendering it highly susceptible to vascular dysfunction. Understanding mechanisms of retinal vascular abnormalities is critical for therapies that preserve vascular and neuronal integrity, yet progress has been hindered by limited models and genetic diversity. To address this gap, we examined the retinal vasculature in multiple aged strains from the BXD recombinant inbred mouse panel, a genetically diverse, tractable, and physiologically relevant platform for uncovering novel genetic drivers and disease mechanisms. We identified BXD32 as a striking outlier with dramatically reduced vessel density. Using optical coherence tomography, optical coherence tomography angiography, and histological analyses, we comprehensively characterized retinal vasculature and structural integrity of BXD32 mice during aging. We found progressive, age-dependent vascular dysfunction and degeneration, beginning in the deep capillary plexus and advancing to the intermediate and superficial layers. These changes were accompanied by neuronal degeneration, including photoreceptor loss and thinning of the ganglion cell complex. Our findings establish BXD32 as a spontaneous and genetically tractable model of inherited retinal neurovascular degeneration and provide a foundation for future studies to identify causative genetic loci and underlying molecular mechanisms.

## 1. Introduction

Retinal vasculature is essential for maintaining visual function by supplying oxygen and nutrients to the retina’s highly active neurons such as retinal ganglion cells (RGCs) and bipolar cells. Unlike other tissues, the retina lacks redundant blood supply, making it particularly susceptible to disruptions in blood flow and vascular integrity [1]. Pathological changes in retinal vessels such as capillary dropout, vessel occlusion, increased permeability, and abnormal neovascularization are characteristics of retinal diseases like diabetic retinopathy [2] and retinal vein occlusion [3]. These vascular alterations can lead to hypoxia, inflammation, neuronal degeneration, and ultimately, vision loss. Increased irregularity in retinal artery diameters and reduced vessel density also occur with aging, contributing to impaired perfusion and heightened vulnerability to retinal diseases [4,5]. Understanding the mechanisms underlying retinal vascular dysfunction/degeneration is important for developing therapeutic strategies aimed at preserving both vascular and neuronal function, ultimately helping to prevent vision loss and improve quality of life in affected individuals. Nonetheless, current research on retinal vascular degeneration is limited by insufficient models and limited genetic variability.

Transcriptome-wide association studies (TWAS) and systems genetics are powerful methodologies for exploring the relationships between specific genes and complex phenotypes, offering valuable insights into gene–trait associations and identifying potential genetic markers for disease susceptibility [6,7]. The BXD family of recombinant inbred mouse strains was derived from a cross between the C57BL/6J (B6) and DBA/2J (D2) parental strains, with substrains subsequently developed through successive breeding strategies, including advanced intercrosses [8,9]. This process yields lines that are nearly genetically fixed but with distinct, recombined mosaics of parental genomes, making BXD strains exceptionally powerful for genetic mapping and systems genetics studies. With over 150 reproducible and genetically diverse substrains [9], the BXD panel offers a genetically diverse, tractable, and physiologically relevant platform to uncover novel genetic drivers, disease modifiers, and mechanistic insights into human diseases [10,11,12,13,14,15]. Recent systems genetics studies using the BXD panel have mapped complex traits ranging from motor coordination and reflex development [16] to metabolic regulation [10], underscoring the versatility of this genetically diverse resource. In addition, BXD strains have been used to identify novel genetic drivers in diverse physiological systems [17,18,19,20], highlighting their broad applicability for uncovering genetic modifiers.

A key requirement for using BXD strains to identify genetic traits is that phenotypic differences must exist among the strains. Therefore, to assess the potential of BXD strains for studying the mechanisms of retinal vascular degeneration during aging, we examined the retinal vasculature in several aged BXD strains. While vessel density was similar across most BXD strains we examined, we unexpectedly identified BXD32 as an outlier, exhibiting a dramatic reduction in vessel density. Therefore, we comprehensively characterized the retinal vasculature and structural integrity in BXD32 mice by employing optical coherence tomography (OCT), OCT angiography (OCTA), and histology at different ages. Unlike conventional BXD lines created directly from B6 × D2 crosses, BXD32 arose through a specific backcross to DBA/2J before sib-mating, resulting in a genetic background that is approximately 75% DBA/2J and 25% C57BL/6J according to Jackson Laboratory strain documentation (Jackson Laboratory Strain 000078) [21]. This atypical breeding history gives BXD32 a distinct haplotype structure and inheritance pattern that enhances its relevance for exploring polygenic contributions to disease [21,22]. In this strain, we discovered age-dependent vascular dysfunction and degeneration, accompanied by neuronal degeneration, highlighting this strain as a promising model for investigating hereditary influences on retinal degeneration.

## 2. Results

### 2.1. Identification of BXD32 as an Outlier with Markedly Reduced Retinal Vessel Density

**Figure 1:**
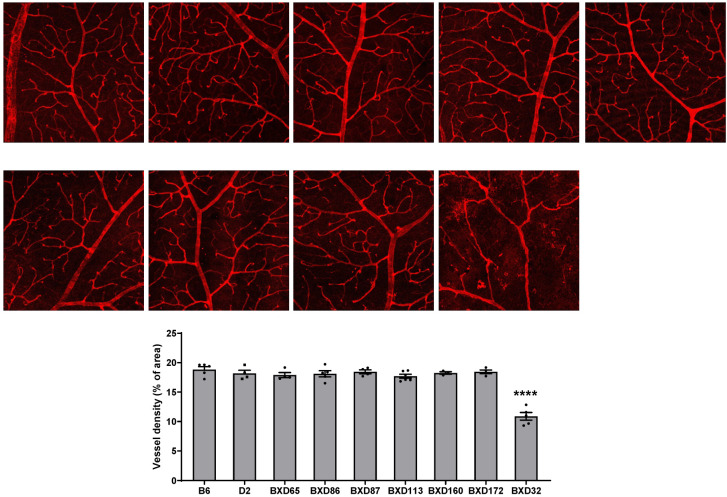
Assessment of retinal vasculature in aged mouse strains. The retinal vasculature of mice older than two years, including B6, D2, and various BXD strains, was examined by staining retinal flatmounts with isolectin B4. Confocal images of the superficial vascular complex (SVC) were taken at 10× magnification, and vessel density was quantified. *n* = 3–6 eyes; **** *p* < 0.0001 compared with all other strains.

To evaluate the suitability of BXD strains for investigating mechanisms of retinal vascular degeneration associated with aging, we stained retinal vessels with isolectin B4 and quantified vessel density of the superficial vascular complex (SVC) in several aged (>2-year-old) BXD strains, along with the parental C57BL/6J (B6) and DBA/2J (D2) strains. Most BXD strains, including BXD65, BXD86, BXD87, BXD113, BXD160, and BXD172, exhibited vessel densities comparable to B6 and D2 or only modest deviations (Figure 1). In contrast, BXD32 strain displayed a striking reduction in retinal vessel density, distinguishing it as a clear outlier among the strains analyzed and warranting further investigation (Figure 1).

### 2.2. Early Retinal Degeneration in Young BXD32 Mice

**Figure 2:**
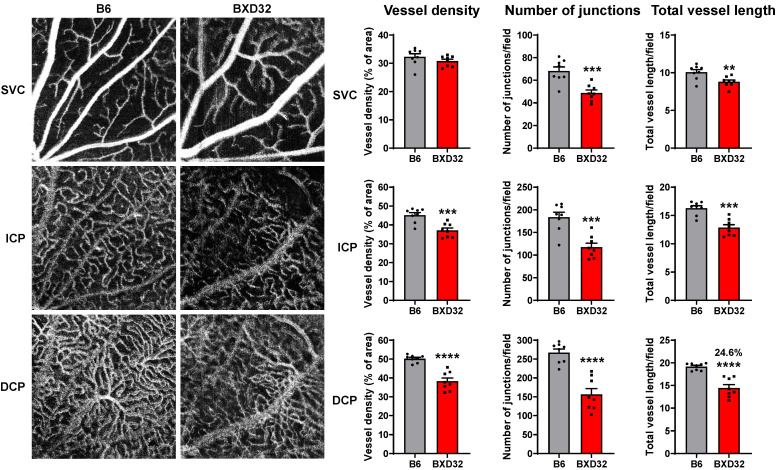
Retinal vascular function is impaired in BXD32 mice at 2 months of age. Representative OCTA images of 2-month-old B6 and BXD32 mice were shown. Vessel density, vessel branch points (junctions) and total vessel length were quantified with AngioTool. *n* = 8 eyes; ns: not significant; ** *p* < 0.01, *** *p* < 0.001, and **** *p* < 0.0001 compared with B6 mice; SVC: superficial vascular complex; ICP: intermediate capillary plexus; DCP: deep capillary plexus.

The dramatic reduction in retinal vessel density observed in aged BXD32 mice prompted us to question whether this reduction resulted from a defect in retinal vascular development or from progressive vascular degeneration during aging. To address this question, we assessed early-stage retinal vascular integrity in 2-month-old mice using OCTA and performed quantitative analysis with AngioTool software version 0.6a to measure vessel density, number of junctions, and total vessel length in the SVC, intermediate capillary plexus (ICP), and deep capillary plexus (DCP) [23]. Unlike in aged BXD32 retinas, vessel density in the SVC was comparable between 2-month-old B6 and BXD32 mice, although significant reductions in the number of vascular junctions and total vessel length were noted in BXD32 retinas (Figure 2, upper panels). In the ICP, BXD32 mice exhibited a pronounced decline in vessel density, number of junctions, and total vessel length relative to B6 mice (Figure 2, middle panels). This pattern of vascular impairment was even more severe in the DCP, where BXD32 mice exhibited highly significant reductions in all three parameters (Figure 2, lower panels). These findings indicate that, by two months of age, vascular impairment in BXD32 mice is most prominent in the deeper retinal capillary networks, with the DCP being the most severely impacted. In contrast, the SVC is relatively less affected, with vessel density remaining intact despite reductions in vascular junctions and total vessel length.

**Figure 3:**
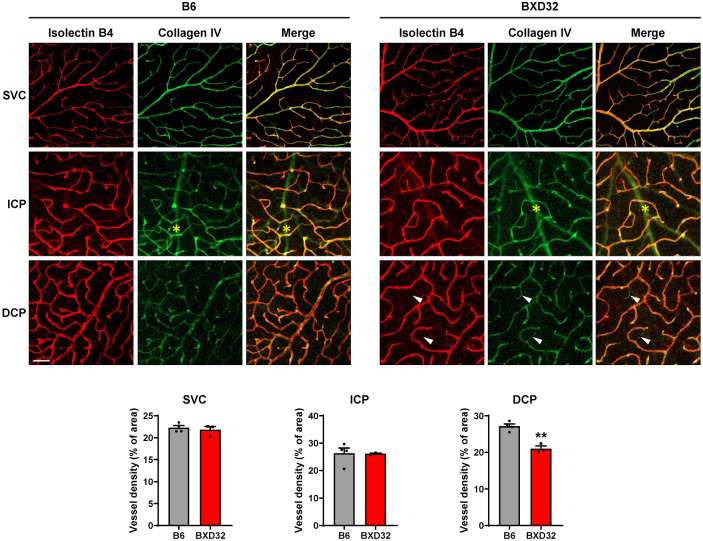
Vascular degeneration occurs in the DCP of BXD32 mice at 2 months of age. Retinal vasculature of 2-month-old B6 and BXD32 mice was examined by staining retinal flatmounts with isolectin B4 (red) and collagen IV (green), and confocal images in the SVC, ICP and DCP were taken at the mid-peripheral retinas. Arrowhead: locations of regressed vessels. *: shadow of the larger retinal vessels from SVC. Vessel density was quantified with AngioTool. Scale bar: 50 μm. *n* = 3–4 eyes; ** *p* < 0.01 compared with B6 mice; SVC: superficial vascular complex; ICP: intermediate capillary plexus; DCP: deep capillary plexus.

As OCTA detects perfused, structurally intact vessels by resolving erythrocyte motion [23], the reduced vessel density observed in BXD32 mice prompted evaluation of whether retinal vasculature undergoes concomitant degeneration with impaired capillary perfusion. Retinas of 2-month-old WT and BXD32 mice were dissected and stained with isolectin B4 to visualize individual vascular layers and antibody against collagen IV, a basement membrane component, to identity “empty collagen IV sleeves” where collagen IV is present but isolectin B4 is absent, indicative of recent vessel regression [24]. Images of the vasculature were captured by confocal microscope (Figure 3). In WT retinas, most vessels in each vascular layer were co-labeled with both isolectin B4 and collagen IV, indicating an intact vasculature. In contrast, BXD32 mice exhibited a greater number of empty collagen IV sleeves, where collagen IV is present but isolectin B4 is absent, particularly in the DCP, suggesting increased capillary degeneration in this layer. Quantitative analysis using AngioTool confirmed a significant reduction in vessel density in the DCP of BXD32 mice, while no significant differences were observed between BXD32 and WT mice in the ICP and SVC. The increased presence of empty collagen IV sleeves in the DCP, but not in the ICP, suggests that capillary regression partially contributes to the reduced vessel density detected by OCTA imaging in the DCP, whereas the reduction in the ICP is likely due to vascular dysfunction. Furthermore, the relatively intact retinal vasculature observed in BXD32 mice at this stage indicates that the dramatic reduction in vessel density seen in aged BXD32 mice is not the result of developmental defects.

**Figure 4:**
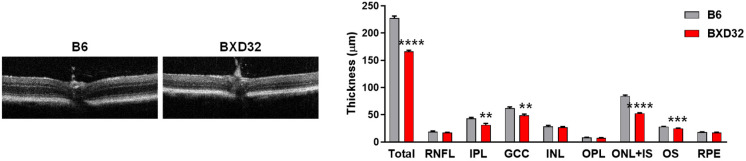
Retinal thickness is reduced in BXD32 mice at 2 months of age. Representative OCT images of 2-month-old B6 and BXD32 mice were shown, and retinal thickness was quantified. *n* = 5–6 eyes; ** *p* < 0.01, *** *p* < 0.001, and **** *p* < 0.0001 compared with B6 mice. Total: Overall retinal thickness; RNFL: Retinal nerve fiber layer; IPL: Inner plexiform layer; GCC: Ganglion cell complex; INL: Inner nuclear layer; OPL: Outer plexiform layer; ONL + IS: Outer nuclear layer plus inner segment; OS: Outer segment; RPE: Retinal pigment epithelium.

Considering the close anatomic and functional association between retinal vessels and neurons, we also assessed retinal neuronal structures by OCT (Supplementary Figure S1). OCT imaging showed that BXD32 mice exhibited significantly thinner retinas than age-matched WT mice (Figure 4). Quantitative analysis revealed a marked reduction in total retinal thickness in BXD32 mice. Layer-specific analysis indicated preferential thinning in the outer nuclear layer plus inner segment (ONL + IS) and outer segment (OS) of photoreceptors, suggesting early photoreceptor degeneration. Significant reductions were also observed in the inner plexiform layer (IPL) and ganglion cell complex (GCC) that includes the retinal nerve fiber layer (RNFL), ganglion cell layer (GCL), and IPL, suggesting early degeneration of inner retinal neurons. Other retinal layers, including the RNFL, INL, OPL and retinal pigment epithelium (RPE) appeared relatively preserved. These findings indicate that BXD32 mice also develop early neuronal degeneration, particularly affecting photoreceptors and ganglion cell-related structures.

### 2.3. Progressive Retinal Degeneration in BXD32 Mice

**Figure 5:**
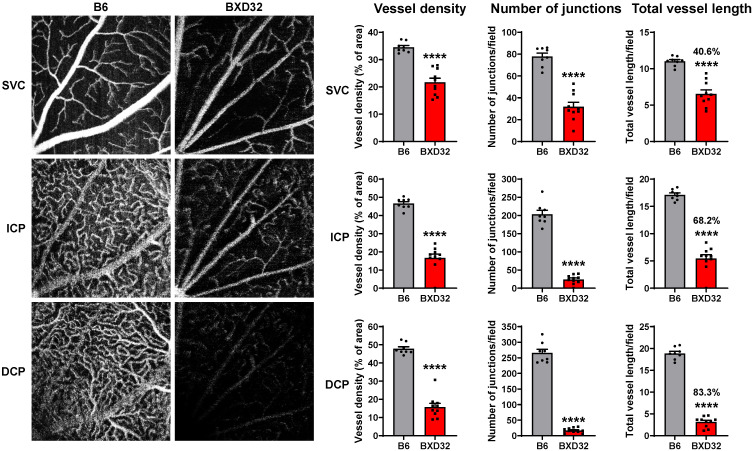
Retinal vascular function is further impaired in BXD32 mice at 8 months of age. Representative OCTA images of 8-month-old B6 and BXD32 mice were shown. Vessel density, vessel branch points (junctions) and total vessel length were quantified with AngioTool. *n* = 8–10 eyes; **** *p* < 0.0001 compared with B6 mice; SVC: superficial vascular complex; ICP: intermediate capillary plexus; DCP: deep capillary plexus.

To evaluate the progressive alterations of retinal structure and vascular integrity in BXD32 strain, we repeated all imaging modalities on a separate cohort of B6 and BXD32 mice at 8 months of age. OCTA further revealed severe, progressive vascular dysfunction in BXD32 mice, characterized by extensive attenuation of vascular perfusion across all three vascular layers (SVC, ICP, and DCP) (Figure 5). In contrast to 2-month-old BXD32 mice, 8-month-old BXD32 retinas exhibited significantly reduced vessel density, number of junctions, and total vessel length in all layers, with a more pronounced decline compared to WT controls (Figure 5 and Supplementary Figure S2). The severity of vascular dysfunction increased with retinal depth, with the most substantial impairment occurring in the DCP (Figure 5 and Supplementary Figure S2). This widespread vascular deterioration suggests an age-dependent worsening of microvascular dysfunction in BXD32 mice, potentially exacerbating retinal hypoxia and neurodegeneration.

**Figure 6:**
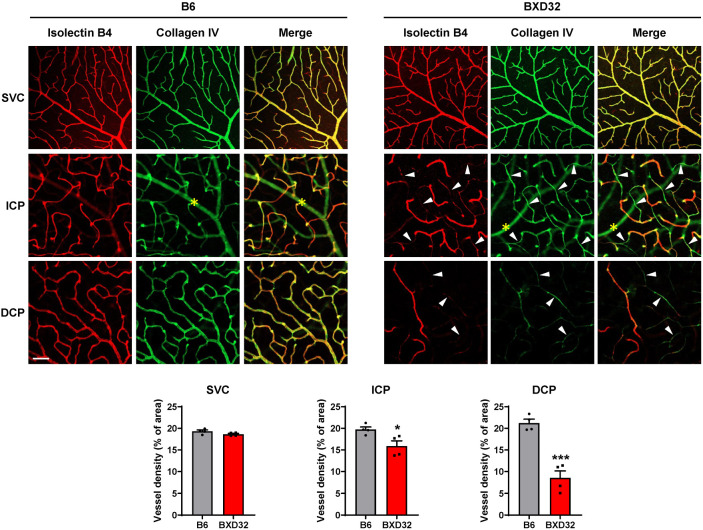
Vascular degeneration occurs in the ICP and DCP of BXD32 mice at 8 months of age. Retinal vasculature of 8-month-old B6 and BXD32 mice was examined by staining retinal flatmounts with isolectin B4 (red) and collagen IV (green), and confocal images in the SVC, ICP and DCP were taken at the mid-peripheral retinas. Arrowhead: locations of regressed vessels. *: shadow of the larger retinal vessels from SVC. Vessel density was quantified with AngioTool. Scale bar: 50 μm. *n* = 4 eyes; * *p* < 0.05 and *** *p* < 0.001 compared with B6 mice; SVC: superficial vascular complex; ICP: intermediate capillary plexus; DCP: deep capillary plexus.

Confocal microscopy findings were consistent with the OCTA results. In 8-month-old BXD32 mice, a significant increase in empty collagen IV sleeves was observed in the retinal microvasculature of both the ICP and DCP, indicating widespread vessel regression (Figure 6). In contrast, large-caliber vessels in the SVC appeared structurally intact, although OCTA imaging revealed that their function was dramatically compromised, suggesting impaired blood flow despite preserved morphology. Compared to the 2-month time point, where vascular degeneration in BXD32 mice was primarily localized to the DCP, 8-month-old BXD32 retinas exhibited extensive vascular regression extending into the ICP, with significantly reduced vessel density in both the ICP and DCP compared to WT mice. These worsening pathologies suggest an age-dependent expansion of vascular degeneration, with capillary loss initially affecting the deeper plexus before advancing to the intermediate vascular layer over time.

**Figure 7:**
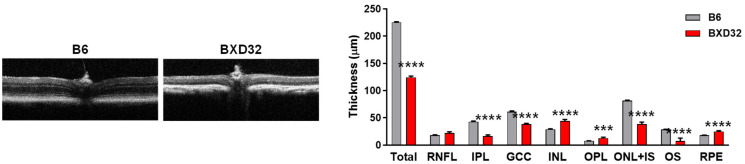
Retinal thickness is further reduced in BXD32 mice at 8 months of age. Representative OCT images of 8-month-old B6 and BXD32 mice were shown, and retinal thickness was quantified. *n* = 4–10 eyes; *** *p* < 0.001 and **** *p* < 0.0001 compared with B6 mice. Total: Overall retinal thickness; RNFL: Retinal nerve fiber layer; IPL: Inner plexiform layer; GCC: Ganglion cell complex; INL: Inner nuclear layer; OPL: Outer plexiform layer; ONL + IS: Outer nuclear layer plus inner segment; OS: Outer segment; RPE: Retinal pigment epithelium.

OCT imaging revealed a marked progression of retinal thinning in BXD32 mice (Figure 7). Total retinal thickness in BXD32 mice was reduced to approximately 50% of that in age-matched B6 mice, with significant thinning observed in retinal layers including the IPL, GCC, ONL + IS and OS layers. This pronounced retinal atrophy suggests ongoing neurodegenerative changes and progressive structural deterioration in BXD32 mice over time.

### 2.4. Severe Retinal and Vascular Degeneration in Aged BXD32 Mice

**Figure 8:**
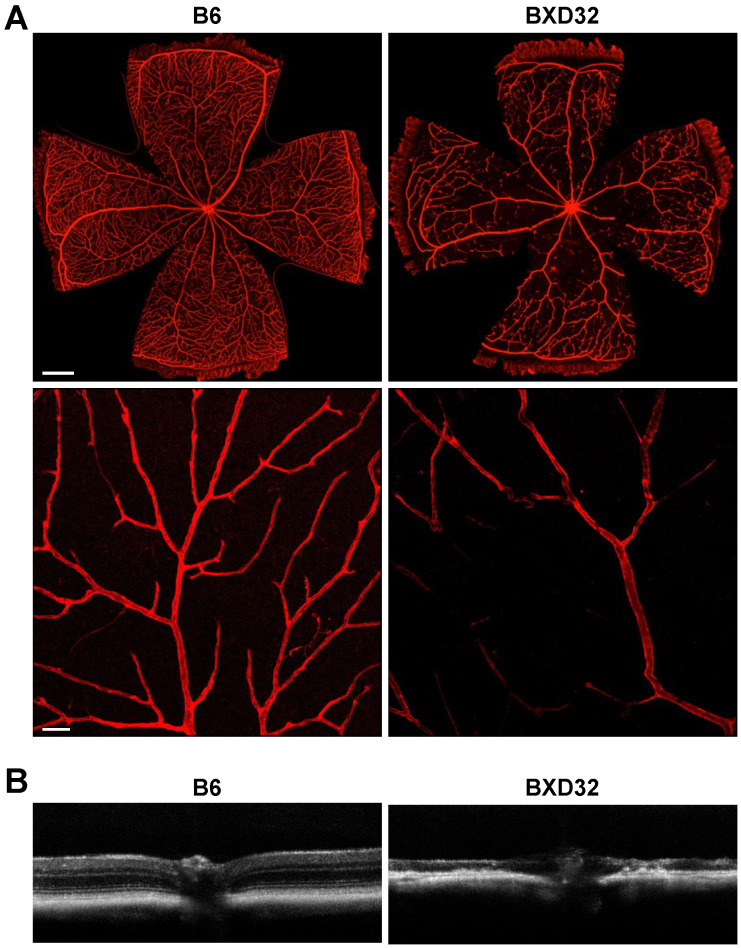
The retinal structure is severely degenerated in BXD32 mice at 2 years of age. (**A**) Retinal vasculature of 2-year-old B6 and BXD32 mice was examined by staining retinal flatmounts with isolectin B4 (red), and confocal images in the superficial vascular complex (SVC) were taken. Scale bar: 500 μm for upper panels and 50 μm for lower panels. *n* = 5 eyes. (**B**) Representative OCT images of 2-year-old B6 and BXD32 mice were shown.

Next, 2-year-old BXD32 and B6 mice were subjected to OCT and confocal microscopy to assess the extent of retinal and vascular degeneration at an advanced age. Dissection and confocal microscopy of BXD32 retinas exhibited progressive and severe retinal vascular degeneration. In contrast to B6 retinas, which displayed an organized and intact vascular network, BXD32 mice exhibited significant vascular obliteration, with the near-complete loss of capillary networks (Figure 8A). Only large-caliber vessels, such as arteries and veins, were preserved, along with a limited number of capillaries restricted to the peripheral retina. Compared to earlier points, where vascular degeneration initially began in the DCP before progressing to the ICP and SVC, 2-year-old BXD32 mice displayed several vascular collapses.

Quantitative OCT measurements could not be obtained in 2-year-old BXD32 mice due to extreme retinal thinning, which disrupted the ability of Bioptigen and Heidelberg Spectralis software version 1.10.4.0 to reliably segment retinal layers and construct accurate images. However, visualization of OCT scans revealed severe retinal thinning in BXD32 mice, with near-total obliteration of all retinal layers compared to B6 mice (Figure 8B).

## 3. Discussion

Many current studies of retinal degeneration utilize transgenic or knockout mouse models, which, while valuable for elucidating specific molecular pathways, may not fully capture the genetic complexity and polygenic nature of most human retinal diseases. The BXD recombinant inbred strain panel provides a genetically diverse resource that more closely mimics the genetic variation found in human populations. These strains serve as a robust resource for investigating polygenic mechanisms that drive complex diseases, such as neurodegenerative disorders [11,12,15], metabolic syndromes [10], and inflammatory pathologies [13,14]. In the context of ophthalmology, BXD mice have contributed to studies on intraocular pressure regulation, RGC loss, and optic nerve degeneration [9]. However, no prior studies have utilized the BXD panel to systematically investigate retinal vascular alterations.

Here, we employed this genetically diverse platform to study the retinal vasculature, leading to the identification of BXD32 as a genetically unique strain exhibiting a spontaneous and progressive form of retinal neurovascular degeneration. While most BXD strains exhibited stable retinal vasculature even at advanced age, BXD32 mice displayed dramatic and age-progressive reductions in vessel density, beginning in the DCP and expanding to the ICP and SVC over time. These vascular changes were accompanied by progressive neurodegeneration, including photoreceptor loss and thinning of the IPL and GCC. Importantly, these changes are unlikely driven by Pde6b (rd1), Crb1 (rd8), or Gnat mutations, as the parental strains (C57BL/6J and DBA/2J) do not carry these alleles. Moreover, intraocular pressure measurements in BXD32 mice did not reveal elevated levels, consistent with previous report [25]. Published work has characterized BXD32 as a spontaneous, polygenic retinal dystrophy model rather than one driven by a single canonical retinal degeneration allele [26].

A few studies have examined BXD32 in the context of various ophthalmic phenotypes. For example, BXD32 mice at 47 days of age have the higher population of RGCs, assessed by counting axons within the optic nerve, compared to other strains and yet they have a very thin photoreceptor layer (Williams et al., 1998, their Figure 4B) [27,28]. Given the known lineage history of subtypes of neurons and photoreceptors [29], this is compatible with a yin-yang balance between numbers of RGC and photoreceptor during development, but it could also be due to differential vascular bed support (or lack thereof) of vulnerable young cells in either deep or superficial layers of the retina after eye opening. Another study identified BXD32 as exhibiting a chronic proinflammatory retinal microenvironment characterized by activation of TNFα, NLRP3 inflammasome, NF-κB, and JAK/STAT signaling pathways, along with increased macrophage activation and phagoptosis [26]. These processes were proposed to act in concert with neuronal loss to exacerbate retinal degeneration.

Our study represents the first to integrate clinically relevant imaging techniques, including OCT and OCTA, with histological analysis to longitudinally assess retinal vasculature in this strain. This approach enabled us to detect early vascular impairment and track its progression over time, building a detailed neurovascular profile of BXD32. OCTA, in particular, offers improved sensitivity and depth-resolved resolution that allowed us to identify early vascular changes in specific retinal layers [23], which were missed using traditional fluorescein angiography [26] and histology. Even as early as 2 months of age, BXD32 retinas exhibited a pronounced decline in vessel density, number of junctions, and total vessel length in the DCP and ICP, and significant reduction in vascular junctions and total vessel length in the SVC, although capillary degeneration was only apparent in the DCP. By integrating data across 2, 8, and 24 months of age, we observed a clear progression of vascular compromise, beginning with early dysfunction in the DCP, followed by degeneration extending into the ICP, and ultimately affecting the SVC in aged animals. Our findings highlight BXD32 not only as a model for retinal dystrophy but also as a powerful tool for studying the reciprocal relationship between vascular degeneration and neurodegeneration in the retina. The primary limitation of OCT imaging in mice is that the small size of the murine eye and thin retinal layers limit the ability to resolve fine details or subtle structural changes. For OCTA, the imaging field is limited, and motion artifacts from respiration and anesthesia can further compromise image quality. We have taken steps to optimize the environment of our imaging procedures such as wrapping mice in cotton gauze during the imaging procedure to restrict movement, utilizing artificial tears throughout the procedure to maintain the transparency of the corneal surface and excluding OCTA images with a quality control rate of 25 decibels or less. We believe our results have high validity and augment the study in a clinically applicable manner.

The precise mechanisms driving retinal vascular dysfunction and degeneration remain to be elucidated. However, given that BXD32 exhibiting a chronic proinflammatory retinal microenvironment with activation of TNFα, NLRP3 inflammasome, NF-κB pathways [26], it is possible that inflammation plays a critical role in the process. NLRP1 and NLRP3 inflammasome activation has been identified in many vascular disorders of the retina including diabetic retinopathy, retinopathy of prematurity, polypoidal choroidal vasculopathy. Notably, inflammasome activation leads to production of IL-1β, which, together with NF-κB signaling, has been shown to damage retinal capillary endothelial cells and increase reactive oxygen species, thereby contributing to microvascular injury [30,31]. Additionally, TNFα is a molecular inducer of apoptosis, causing loss of retinal microvascular cells with resultant vascular permeability [31]. For these reasons, it is not surprising that we observed vascular dysfunction in the DCP in BXD-32 mice.

Of note, while we demonstrate both vascular and neurodegenerative changes in BXD32 mice, we did not perform functional assessments such as electroretinography (ERG) or pattern ERG, and thus we were unable to directly correlate structural alterations with visual function. Nevertheless, prior human and experimental studies have demonstrated strong correlations between retinal structural changes and functional decline in many diseases including diabetic retinopathy [32], glaucoma [33,34,35], neurodegenerative disease [36] and retinitis pigmentosa [37,38,39], lending support to our interpretation. Future work incorporating functional assays will be needed to directly test these relationships and to further validate BXD32 as a model of progressive retinal degeneration. In addition, while we identified progressive and dramatic retinal vascular dysfunction and degeneration in BXD32, setting the stage for using the BXD panel to identify polygenic mechanisms underlying vascular injury in the retina, additional work is necessary to achieve this goal. One promising approach involves generating an N2 population by crossing the BXD32 strain with B6 to produce F1 hybrids, followed by a backcross of these F1s to BXD32. This design enriches for BXD32 alleles while maintaining informative segregation from B6, enabling high-resolution quantitative trait locus (QTL) mapping. Because all BXD strains have been fully genotyped, sparse marker data from N2 offspring can be used to infer genome-wide haplotypes, facilitating the identification of genetic loci associated with retinal vascular traits. Additionally, expanding the retinal vascular phenotyping to a broader set of BXD strains could reveal additional models with distinct or overlapping pathologies, further enriching our capability to use the BXD approach to understand the genetic contributors to retinal vascular degeneration.

In summary, we identified BXD32 mice as a spontaneous and genetically tractable model of progressive retinal neurovascular degeneration. These findings establish a foundation for future genetic mapping studies aimed at identifying causative loci and molecular mechanisms, which may ultimately inform therapeutic strategies for human retinal vascular diseases. By characterizing progressive retinal vascular dysfunction and degeneration in this strain for the first time, our study fills a critical knowledge gap and paves the way for broader applications of BXD systems genetics in ophthalmic disease research.

## 4. Materials and Methods

### 4.1. Animals

Aged BXD recombinant inbred mouse lines were bred and maintained in Dr. Lu Lu’s laboratory at the University of Tennessee Health Science Center [25]. C57BL/6J (B6) (#000664) mice and BXD32 (#000078) mice were originally obtained from the Jackson Laboratory (Bar Harbor, ME, USA) and bred at the University of Texas Medical Branch Animal Resource Center to generate subsequent offspring generations. Both male and female mice were used in all experiments, with no apparent sex-related differences observed. Mice were housed under a 12 h light/dark cycle with ad libitum access to food and water. All experimental procedures were approved by the Institutional Animal Care and Use Committee (IACUC) at the University of Texas Medical Branch and the University of Tennessee Health Science Center and conducted in accordance with the Association for Research in Vision and Ophthalmology (ARVO) Statement for the Use of Animals in Ophthalmic and Vision Research.

### 4.2. Optical Coherence Tomography (OCT) Imaging

B6 and BXD32 mice underwent OCT imaging at 2, 8, and 24 months of age prior to sacrifice, as previously described [40]. Mice were sedated with an intraperitoneal injection of xylazine (10 mg/kg) and ketamine (100 mg/kg). Pupils were subsequently dilated with one drop each of tropicamide and phenylephrine. OCT imaging was performed using a Spectral Domain Ophthalmic Imaging System (Envisu R2200, Bioptigen Inc., Durham, NC, USA). Three successive OCT scans were captured for each retina, consisting of 1000 A-scans and 100 B-scans. Each scan covered a donut-shaped region surrounding the optic nerve head in order to remove variance of the optic nerve head during quantitative analysis. The inner radius was 200 μm and the outer radius 700 μm from the center of the optic nerve disk. Retinal thickness measurements were obtained using the Bioptigen automated report for murine eyes and calculated for each individual retinal layer, as well as for total retinal thickness. The ganglion cell complex (GCC) includes the retinal nerve fiber layer (RNFL), ganglion cell layer (GCL), and inner plexiform layer (IPL). Reported values for total retinal thickness and individual retinal layer represent the mean of three successive scans for each retina. Examiners were blinded to mouse strains during image acquisition and analysis to ensure unbiased measurements.

### 4.3. Optical Coherence Tomography Angiography (OCTA) Imaging

B6 and BXD32 mice underwent OCTA imaging at 2 and 8 months of age as previously described [23]. Mice were first sedated using the Digital Low-Flow Anesthesia System (Kent Scientific, Torrington, CT, USA) with a 3.8% isoflurane infusion. One drop each of phenylephrine and tropicamide was administered to both eyes to induce mydriasis. Mice were subsequently immobilized by wrapping in cotton gauze and placed on an imaging platform with a continuous supply of isoflurane to minimize the risk of cataract formation during OCTA imaging caused by prolonged sedation. To prevent corneal desiccation, GenTeal^®^ Tears was applied to the non-imaged eye throughout the procedure. OCTA images were acquired using the Heidelberg HRA + OCT Spectralis (Heidelberg Engineering, Heidelberg, Germany). A 20° by 20° high resolution scan was acquired for the superotemporal, inferotemporal, superonasal, and inferonasal quadrants of each retina, with the optic nerve head positioned in the corner. OCTA scans with a quality-control index of 25 decibels or less were excluded from further analysis. Each OCTA image was analyzed using Angiotool software version 0.6a (National Cancer Institute, Bethesda, MD, USA) to quantify vessel density, the number of branch points, and total vessel length [41]. The reported values for each retina represent the mean across all imaged quadrants. Throughout image acquisition and analysis, examiners were blinded to mouse strains to ensure unbiased results.

### 4.4. Retinal Dissection and Flatmounts

B6 and BXD32 mice were sacrificed at 2 months, 8 months, and 2 years of age. Eyeballs were enucleated and fixed in 4% paraformaldehyde (PFA) at 4 °C overnight [40]. Similar procedures were applied to D2 and other BXD strains. The retinas were then dissected away from the retinal pigmented epithelium (RPE) and choroid, followed by PBS washes, blocking and permeabilization in PBS containing 5% donkey serum and 0.3% Triton X-100 (Thermo Fisher Scientific, Waltham, MA, USA) for 3 h at room temperature. Retinas were then incubated with Alexa Fluor 568-conjugated isolectin B4 (1:200, Thermo Fisher Scientific) and anti-collagen IV (1:200, Southern Biotech, AL, USA) at 4 °C overnight, followed with secondary antibody. Finally, retinas were mounted on glass slides and imaged using confocal microscopy (LSM 800, Carl Zeiss Inc., Thornwood, NY, USA). Each final exported image was generated from a stack of several optical slices. Vessel regression was assessed by identifying collagen IV–positive, isolectin B4–negative basement membrane sleeves [24].

### 4.5. Statistical Analysis

For all analyses, the mean and standard error of the mean (SEM) were calculated. To compare WT and BXD32 mice, a Student’s *t*-test was performed using GraphPad Prism software version 10.4.2 (GraphPad Software Inc., La Jolla, CA, USA). A *p*-value < 0.05 was considered statistically significant.

## Data Availability

All data needed to evaluate the conclusions in the paper are present in the paper.

## Supplementary Materials

The supporting information can be downloaded at: https://www.mdpi.com/article/10.3390/ijms26199289/s1.
