# Supplementary material for: Progressive Retinal Vascular and Neuronal Degeneration in BXD32 Mice: A Model for Age-Dependent Neurovascular Pathology
Source: Int J Mol Sci. 2025 Sep 23;26(19):9289. doi: 10.3390/ijms26199289 (PMC12525183; doi:10.3390/ijms26199289)
Supplement: Supplementary file 1 [file ijms-26-09289-s001.zip › ijms-3822889-supplementary.pdf]

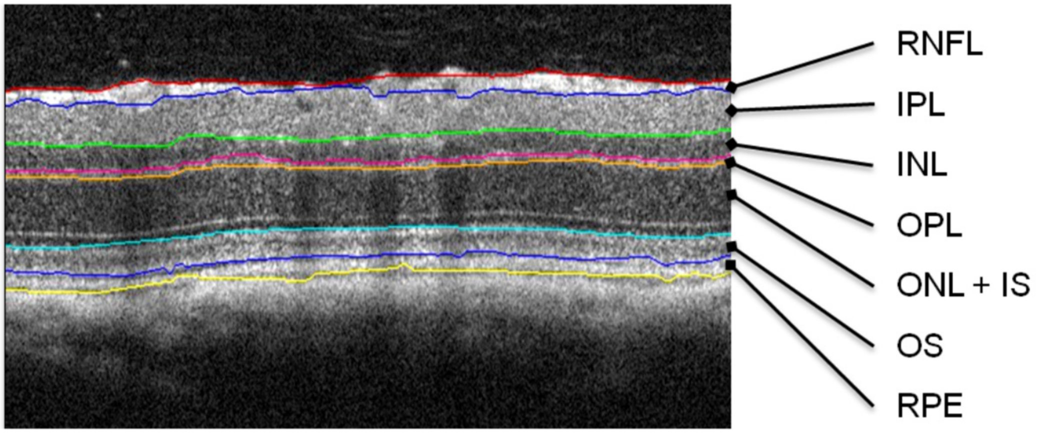

Supplementary Figure S1. Representative OCT image of mouse retina. Segmentation lines for individual retinal layers were generated using the Bioptigen automated report for murine eyes. RNFL: Retinal nerve fiber layer; IPL: Inner plexiform layer; INL: Inner nuclear layer; OPL: Outer plexiform layer; ONL+IS: Outer nuclear layer plus inner segment; OS: Outer segment; RPE: Retinal pigment epithelium.

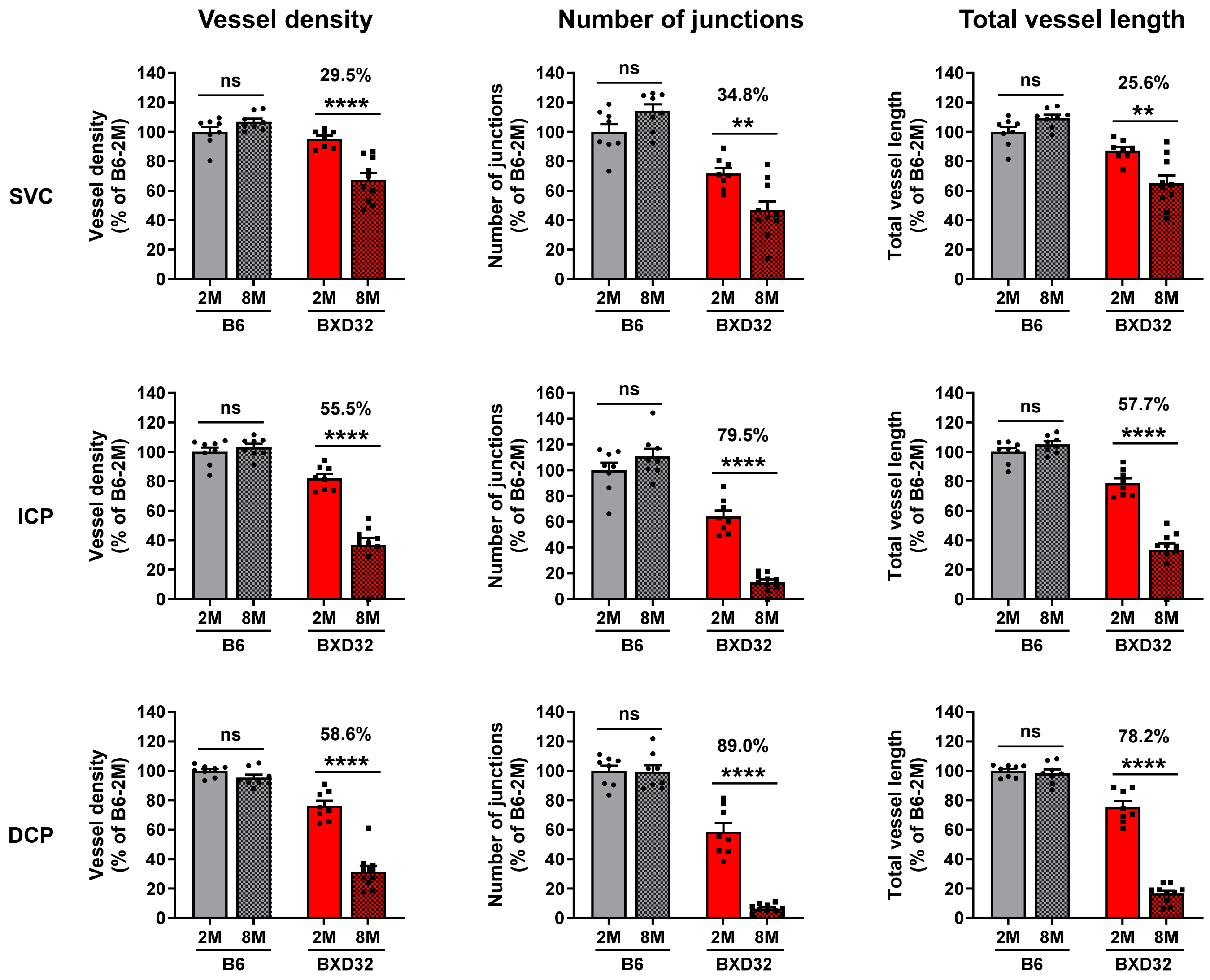

Supplementary Figure S2. Age-dependent impairment of retinal vascular function in BXD32 mice. Longitudinal statistical analyses of OCTA images from B6 and BXD32 retinas at 2 and 8 months of age were performed to assess vessel density, vessel junctions, and total vessel length. Values were normalized to those of 2-month-old B6 mice. n=8-10 eyes; ns: not significant; **p<0.01, ****p<0.0001. SVC: superficial vascular complex; ICP: intermediate capillary plexus; DCP: deep capillary plexus.
